# Mouse optical imaging for understanding resting-state functional connectivity in human fMRI

**DOI:** 10.1080/19420889.2018.1528821

**Published:** 2018-10-21

**Authors:** Teppei Matsui, Tomonari Murakami, Kenichi Ohki

**Affiliations:** a Department of Physiology, The University of Tokyo School of Medicine, Tokyo, Japan; b International Research Center for Neurointelligence (WPI-IRCN), The University of Tokyo, Tokyo, Japan

**Keywords:** fMRI, calcium imaging, mouse, functional connectivity, resting-state

## Abstract

Resting-state functional connectivity (FC), which measures the temporal correlation of spontaneous hemodynamic activity between distant brain areas, is a widely accepted method in functional magnetic resonance imaging (fMRI) to assess the connectome of healthy and diseased human brains. A common assumption underlying FC is that it reflects the temporal structure of large-scale neuronal activity that is converted into large-scale hemodynamic activity. However, direct observation of such relationship has been difficult. In this commentary, we describe our recent progress regarding this topic. Recently, transgenic mice that express a genetically encoded calcium indicator (GCaMP) in neocortical neurons are enabling the optical recording of neuronal activity in large-scale with high spatiotemporal resolution. Using these mice, we devised a method to simultaneously monitor neuronal and hemodynamic activity and addressed some key issues related to the neuronal basis of FC. We propose that many important questions about human resting-state fMRI can be answered using GCaMP expressing transgenic mice as a model system.

Temporal correlation of spontaneous hemodynamic signals, commonly referred to as FC [1], is one of the most widely used fMRI methods to study functional network organization of the human brain in a non-invasive manner. The overall pattern of FC closely resembles anatomical connectivity [2] as well as effective connectivity assessed with electrical microstimulation [3,4]. Moreover, recent studies suggest that FC is sensitive enough to detect network-level functional changes due to behavioral training [5], wakefulness levels [6] and psychiatric diseases [7]. A key assumption underlying FC is that it reflects the large-scale spatiotemporal dynamics of spontaneous neuronal activity [8]. For appropriate interpretation of FC, however, we must verify its neuronal basis

Several groups have recently used transgenic mice expressing a genetically encoded calcium indicator (GCaMP) in neocortical neurons [9] to simultaneously observe neuronal calcium and optical intrinsic signals that reflect hemodynamic activity (Figure 1(a)). Some groups including ours monitored neuronal and hemodynamic activity in the entire dorsal neocortex of the transgenic mice using green (530 nm) and red (630 nm) channels, respectively [10–12]. At 630 nm, the optical intrinsic signal primarily reflects the deoxyhemoglobin signal [13]. Other groups have incorporated additional wavelengths to further disambiguate different hemodynamic components [14,15].

Using this method, we examined the relationship between fast spatiotemporal patterns of neuronal calcium activity and the spatial pattern of FC. We found a significant relationship between two seemingly different types of large-scale spontaneous neuronal activities – namely, global waves propagating across the neocortex and transient coactivations among cortical areas sharing high FC. Different sets of cortical areas, sharing high FC within each set, were coactivated at different timings of the propagating global waves, suggesting that spatial information of cortical network characterized by FC was embedded in the phase of the global waves (Figure 1(b)). Furthermore, we confirmed that such transient coactivations in calcium signal were converted into spatially similar coactivations in hemodynamic signal and were necessary to sustain the spatial structure of FC measured in hemodynamic signal. The present method also revealed that the conversion of the spatial pattern of neuronal activity to that of hemodynamic activity was not perfect. The accuracy of conversion between the spatial pattern of neuronal activity and that of hemodynamic activity was modulated as a function of the strength of the vascular signal and neuronal activity. In the presence of strong vascular signal or when neuronal activity was weak, the spatial pattern of neuronal activity was not faithfully converted into that of hemodynamic activity (Figure 1(c)). Together, these results explain how global waves of spontaneous neuronal activity propagating across large-scale cortical network contribute to FC measured in hemodynamic signal in the resting state.

**Figure 1.**
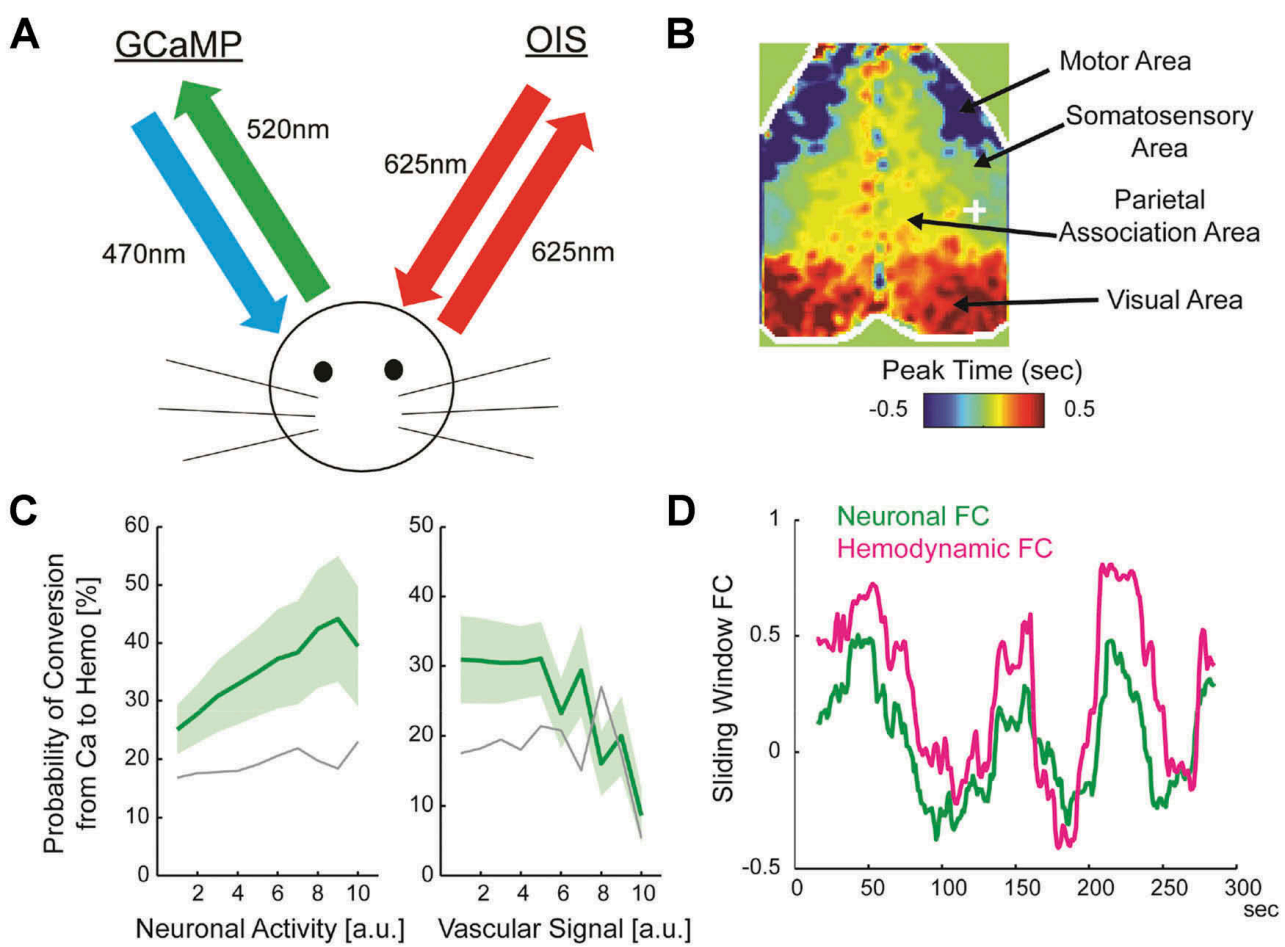
Simultaneous imaging of calcium and hemodynamic signals to investigate neuronal basis of resting-state FC.(a) Experimental setup. Neuronal activity is monitored with GCaMP fluorescence. Hemodynamic activity is monitored simultaneously with GCaMP by means of optical intrinsic signal. (b) Spatial organization of FC is encoded in the phase of globally propagating spontaneous neuronal activity. Phase is calculated relative to a region-of-interest indicated by white cross. (c) Probability of conversion from spatial pattern of neuronal Ca^2+^ signal to that of hemodynamics depends the strength of neuronal activity (left) and non-neuronal physiological noise as measured by the signal in large blood vessels (right). See [10] for details. (d) An example data showing similar temporal fluctuations of dynamic FC (30sec sliding-window) calculated using neuronal activity and dynamic FC calculated using hemodynamic activity.

The proposed method also allows us to ask questions related to newly developed analyses methods for FC in human fMRI [16]. In contrast with the traditional analysis of “static” FC using many minutes of scans, the temporal fluctuation of FC across short time windows gives the dynamic aspect of FC that could provide information on the functional organizations of healthy and diseased brains that is inaccessible with static FC [17–19]. The presence of temporal fluctuations in FC has also influenced theoreticians to constrain realistic models of brain networks [20–22]. However, it is unclear whether the fluctuations of FC measured in hemodynamics reflect the dynamics of underlying neural activity. Using simultaneous imaging of neuronal calcium and hemodynamic signals in transgenic mice, we found that the dynamics of FC calculated using hemodynamic signals closely resembled those calculated using calcium signals, suggesting a neuronal origin of the temporal fluctuations of hemodynamic FC (Figure 1(d)). Moreover, temporal fluctuations of spatial patterns across different short time windows were similar between FC calculated using neuronal calcium signal and FC calculated using hemodynamic signal.

The temporal fluctuation of FC across short time-windows does not necessarily indicate non-stationary FC. Indeed, recent studies using resting-state fMRI in humans reported that the temporal fluctuation of FC cannot be distinguished from that in a model assuming stationary FC and statistical sampling error [23,24]. Applying the same analysis to the mouse data, we found that, in both neuronal calcium and hemodynamic signals, the temporal dynamics of FC were not fully explained by stationary FC [16]. The difference may be attributed to the superior signal-to-noise ratio of mouse data, for both calcium and optical intrinsic signals, compared to human fMRI. Alternatively, the use of anesthetized preparation could have caused non-stationary FC in the mouse data. A recent report using simultaneous measurement of electrophysiological and optical intrinsic signals in local population of mouse somatosensory neurons showed that “spontaneous” hemodynamic activity is driven by behavior (e.g., whisking) and correlate only weakly with neuronal activity [25]. Whether or not such “spontaneous” hemodynamic activity contains spatial patterns relevant to resting-state FC remains unclear. An alternative possibility is that such “spontaneous” hemodynamic activity is global and thus mostly removed by the global signal regression typically used in the resting-state FC analysis. Future experiments using large-scale imaging in awake mice are needed to clarify these points.

There are several future applications of the proposed method. For example, resting-state FC in mouse models of mental diseases can be used to examine the possibility of diagnosis based on resting-state FC [26]. Genetic tools in mice also allows monitoring of cell-type and cortical layer-dependent neuronal activity [27], which is difficult in other model species such as primates [28]. Furthermore, because GCaMP mice allow monitoring of neuronal activity even at very early stages of development [29,30], developmental changes of resting-state FC can be tracked at a high signal-to-noise ratio. In summary, combined calcium and optical intrinsic signal imaging using GCaMP transgenic mice is becoming a powerful platform to clarify key issues in human fMRI.

## References

[1] Fox MD, Raichle ME. Spontaneous fluctuations in brain activity observed with functional magnetic resonance imaging. Nat Rev Neurosci. 2007;8(9):700–711. PubMed PMID: 17704812 17704812 10.1038/nrn2201

[2] Honey CJ, Sporns O, Cammoun L, et al Predicting human resting-state functional connectivity from structural connectivity. Proc Natl Acad Sci USA. 2009;106(6):2035–2040. Epub 2009/02/02. 19188601 10.1073/pnas.0811168106 PMC2634800

[3] Matsui T, Tamura K, Koyano KW, et al Direct comparison of spontaneous functional connectivity and effective connectivity measured by intracortical microstimulation: an fMRI study in macaque monkeys. Cereb Cortex. 2011;21(10):2348–2356. Epub 2011/ 03/02. 21368090 10.1093/cercor/bhr019

[4] Matsui T, Koyano KW, Tamura K, et al FMRI activity in the macaque cerebellum evoked by intracortical microstimulation of the primary somatosensory cortex: evidence for polysynaptic propagation. PLoS One. 2012;7(10):e47515 Epub 2012/ 10/31. 23118875 10.1371/journal.pone.0047515 PMC3485272

[5] Lewis CM, Baldassarre A, Committeri G, et al Learning sculpts the spontaneous activity of the resting human brain. Proc Natl Acad Sci USA. 2009;106(41):17558–17563. Epub 2009/10/05. 19805061 10.1073/pnas.0902455106 PMC2762683

[6] Tagliazucchi E, Laufs H. Decoding wakefulness levels from typical fMRI resting-state data reveals reliable drifts between wakefulness and sleep. Neuron. 2014;82(3):695–708. PubMed PMID: 24811386. 24811386 10.1016/j.neuron.2014.03.020

[7] Calhoun VD, Eichele T, Pearlson G Functional brain networks in schizophrenia: a review. Front Hum Neurosci. 2009;3:17 PubMed PMID: 19738925; PubMed Central PMCID: PMCPMC2737438 Epub 2009/ 08/17. 19738925 10.3389/neuro.09.017.2009 PMC2737438

[8] Leopold DA, Maier A Ongoing physiological processes in the cerebral cortex. Neuroimage. 2012;62(4):2190–2200. Epub 2011/ 10/25. 22040739 10.1016/j.neuroimage.2011.10.059 PMC3288739

[9] Zariwala HA, Borghuis BG, Hoogland TM, et al A Cre-dependent GCaMP3 reporter mouse for neuronal imaging in vivo. J Neurosci. 2012;32(9):3131–3141. PubMed PMID: 22378886; PubMed Central PMCID: PMCPMC3315707. 22378886 10.1523/JNEUROSCI.4469-11.2012 PMC3315707

[10] Matsui T, Murakami T, Ohki K Transient neuronal coactivations embedded in globally propagating waves underlie resting-state functional connectivity. Proc Natl Acad Sci USA. 2016;113(23):6556–6561. PubMed PMID: 27185944. 27185944 10.1073/pnas.1521299113 PMC4988587

[11] Vanni MP, Murphy TH Mesoscale transcranial spontaneous activity mapping in GCaMP3 transgenic mice reveals extensive reciprocal connections between areas of somatomotor cortex. J Neurosci. 2014;34(48):15931–15946. PubMed PMID: 25429135. 25429135 10.1523/JNEUROSCI.1818-14.2014 PMC6608481

[12] Murphy MC, Chan KC, Kim SG, et al Macroscale variation in resting-state neuronal activity and connectivity assessed by simultaneous calcium imaging, hemodynamic imaging and electrophysiology. Neuroimage Epub 2017/ 12/22 2018;169:352–362. PubMed PMID: 29277650; PubMed Central PMCID: PMCPMC5856618. 29277650 10.1016/j.neuroimage.2017.12.070 PMC5856618

[13] Ma Y, Shaik MA, Kim SH, et al Wide-field optical mapping of neural activity and brain haemodynamics: considerations and novel approaches. Philos Trans R Soc Lond B Biol Sci. 2016;371(1705). PubMed PMID: 27574312; PubMed Central PMCID: PMCPMC5003860 DOI:10.1098/rstb.2015.0360 PMC5003860 27574312

[14] Ma Y, Shaik MA, Kozberg MG, et al Resting-state hemodynamics are spatiotemporally coupled to synchronized and symmetric neural activity in excitatory neurons. Proc Natl Acad Sci USA. 2016;113(52):E8463–E71. Epub 2016/12/14. 27974609 10.1073/pnas.1525369113 PMC5206542

[15] Mitra A, Kraft A, Wright P, et al Spontaneous infra-slow brain activity has unique spatiotemporal dynamics and laminar structure. Neuron. 2018;98(2):297–305.e6. Epub 2018/03/29. 29606579 10.1016/j.neuron.2018.03.015 PMC5910292

[16] Matsui T, Murakami T, Ohki K Neuronal origin of the temporal dynamics of spontaneous BOLD activity correlation. Cereb Cortex. 2018 Epub 2018/03/07 PubMed PMID: 29522092 DOI:10.1093/cercor/bhy045. 29522092

[17] Hutchison RM, Womelsdorf T, Allen EA, et al Dynamic functional connectivity: promise, issues, and interpretations. Neuroimage. 2013;80:360–378. Epub 2013/ 05/24 PubMed PMID: 23707587; PubMed Central PMCID: PMCPMC3807588. 23707587 10.1016/j.neuroimage.2013.05.079 PMC3807588

[18] Allen EA, Damaraju E, Plis SM, et al Tracking whole-brain connectivity dynamics in the resting state. Cereb Cortex. 2014;24(3):663–676. Epub 2012/ 11/11. 23146964 10.1093/cercor/bhs352 PMC3920766

[19] Zalesky A, Fornito A, Cocchi L, et al Time-resolved resting-state brain networks. Proc Natl Acad Sci U S A. 2014;111(28):10341–10346. Epub 2014/06/30. 24982140 10.1073/pnas.1400181111 PMC4104861

[20] Hansen EC, Battaglia D, Spiegler A, et al Functional connectivity dynamics: modeling the switching behavior of the resting state. Neuroimage Epub 2014/ 11/10 2015;105:525–535. PubMed PMID: 25462790. 25462790 10.1016/j.neuroimage.2014.11.001

[21] Messé A, Rudrauf D, Benali H, et al Relating structure and function in the human brain: relative contributions of anatomy, stationary dynamics, and non-stationarities. PLoS Comput Biol. 2014;10(3):e1003530 Epub 2014/ 03/20. 24651524 10.1371/journal.pcbi.1003530 PMC3961181

[22] Deco G, Ponce-Alvarez A, Mantini D, et al Resting-state functional connectivity emerges from structurally and dynamically shaped slow linear fluctuations. PubMed PMID: 23825427; PubMed Central PMCID: PMCPMC3718368 J Neurosci. 2013;33(27):11239–11252. 23825427 10.1523/JNEUROSCI.1091-13.2013 PMC3718368

[23] Laumann TO, Snyder AZ, Mitra A, et al On the stability of BOLD fMRI correlations. Cereb Cortex. 2016 Epub 2016/ 09/02 PubMed PMID: 27591147 DOI:10.1093/cercor/bhw265 PMC6248456 27591147

[24] Hindriks R, Adhikari MH, Murayama Y, et al Can sliding-window correlations reveal dynamic functional connectivity in resting-state fMRI? Neuroimage. 2016;127:242–256. Epub 2015/ 11/26 PubMed PMID: 26631813; PubMed Central PMCID: PMCPMC4758830. 26631813 10.1016/j.neuroimage.2015.11.055 PMC4758830

[25] Winder AT, Echagarruga C, Zhang Q, et al Weak correlations between hemodynamic signals and ongoing neural activity during the resting state. Nat Neurosci. 2017;20(12):1761–1769. Epub 2017/ 11/06. 29184204 10.1038/s41593-017-0007-y PMC5816345

[26] Busche MA, Kekuš M, Adelsberger H, et al Rescue of long-range circuit dysfunction in Alzheimer’s disease models. Nat Neurosci. 2015;18(11):1623–1630. Epub 2015/ 10/12. 26457554 10.1038/nn.4137

[27] Madisen L, Garner AR, Shimaoka D, et al Transgenic mice for intersectional targeting of neural sensors and effectors with high specificity and performance. Neuron. 2015;85(5):942–958. PubMed PMID: 25741722; PubMed Central PMCID: PMCPMC4365051. 25741722 10.1016/j.neuron.2015.02.022 PMC4365051

[28] Koyano KW, Takeda M, Matsui T, et al Laminar module cascade from layer 5 to 6 implementing cue-to-target conversion for object memory retrieval in the primate temporal cortex. Neuron. 2016;92(2):518–529. Epub 2016/10/06. 27720482 10.1016/j.neuron.2016.09.024

[29] Murakami T, Matsui T, Ohki K Functional segregation and development of mouse higher visual areas. J Neurosci. 2017;37(39):9424–9437. Epub 2017/08/28. 28847805 10.1523/JNEUROSCI.0731-17.2017 PMC6596770

[30] Ackman JB, Burbridge TJ, Crair MC Retinal waves coordinate patterned activity throughout the developing visual system. Nature. 2012;490(7419):219–225. PubMed PMID: 23060192; PubMed Central PMCID: PMCPMC3962269. 23060192 10.1038/nature11529 PMC3962269

